# Open Excision of a Broad-Based Duodenal Lipoma Not Amenable to Endoscopic Resection: A Case Report

**DOI:** 10.7759/cureus.114774

**Published:** 2026-08-19

**Authors:** Mohamad El Haress, Mohamad I Siblini, Mohammed S Zaatari

**Affiliations:** 1 Department of Surgery, American University of Beirut Medical Center, Beirut, LBN; 2 General Surgery, Rafik Hariri University Hospital, Beirut, LBN; 3 General Surgery, Makassed General Hospital, Beirut, LBN

**Keywords:** duodenal lipoma, duodenotomy, endoscopic ultrasound, submucosal tumor, transduodenal excision

## Abstract

Duodenal lipomas are rare, benign, fat-containing submucosal tumors that are usually silent but can cause bleeding, obstruction, or intussusception once they exceed 2 cm. We report the case of a 40-year-old man with hypertension who presented with postprandial colicky abdominal pain, recurrent melena, and a 10-kg weight loss, having restricted himself to a pureed-food diet for three months. Endoscopy showed a 4-5 cm, broad-based polyp obstructing 75% of the duodenal lumen at the distal first/proximal second portion; forceps biopsy showed only hyperplastic mucosa. Endoscopic ultrasound (EUS) and CT demonstrated a homogeneous, fat-density submucosal mass consistent with lipoma. Given its size and broad base, endoscopic resection was considered unsafe, and the lesion was excised through duodenotomy, preserving the ampulla, bile and pancreatic ducts, and duodenal wall. Histopathology confirmed a submucosal lipoma. The patient recovered uneventfully, with resolution of anemia and symptoms. This report highlights the importance of individualized decision-making when a duodenal lipoma is not amenable to endoscopic resection.

## Introduction

Duodenal lipomas are rare, benign mesenchymal tumors composed of mature adipose tissue, accounting for approximately 4% of benign small-bowel tumors and 20% of benign duodenal neoplasms [1,2]. About 90% of these tumors arise in the submucosa, with most patients diagnosed between 40 and 70 years of age, and approximately 39% of lesions located in the second portion of the duodenum (D2) [1]. Lesions smaller than 2 cm are usually asymptomatic, whereas larger tumors may ulcerate, bleed, cause obstruction, or, in rare cases, intussuscept [3]. There are no convincing reports of malignant transformation in duodenal lipomas. Some authors have suggested a theoretical risk for lesions larger than 5 cm, extrapolated from soft-tissue sarcoma guidelines; however, these guidelines are not based on lipoma- or duodenum-specific data [3,4].

Recent advances in esophagogastroduodenoscopy (EGD) and cross-sectional imaging have led to increased incidental detection of duodenal lipomas. However, preoperative differentiation of lipomas from other subepithelial tumors, including Brunner gland hamartoma, gastrointestinal stromal tumors, ectopic pancreas, and neuroendocrine tumors, based solely on endoscopic appearance remains challenging and generally requires endoscopic ultrasound (EUS) [5,6]. Two endoscopic features aid in this assessment: a positive “pillow sign,” in which the lesion indents and slowly re-expands when probed with closed biopsy forceps, reflecting its soft, fatty consistency; and a broad base, defined as an attachment or stalk width exceeding 1 cm, which helps distinguish it from the narrow, pedunculated stalk typical of lesions amenable to snare resection. Endoscopic submucosal dissection (ESD) and endoscopic full-thickness resection (EFTR) allow for minimally invasive removal of many duodenal lipomas; however, large, broad-based, or sessile lesions may exceed the technical limits of endoscopy and may require surgical resection [7,8,9,10]. We discuss a case of a large, broad-based duodenal lipoma that was not amenable to endoscopic removal and was treated by open transduodenal excision, highlighting the diagnostic rationale and operative approach that enabled successful resection.

## Case presentation

**Figure 1:**
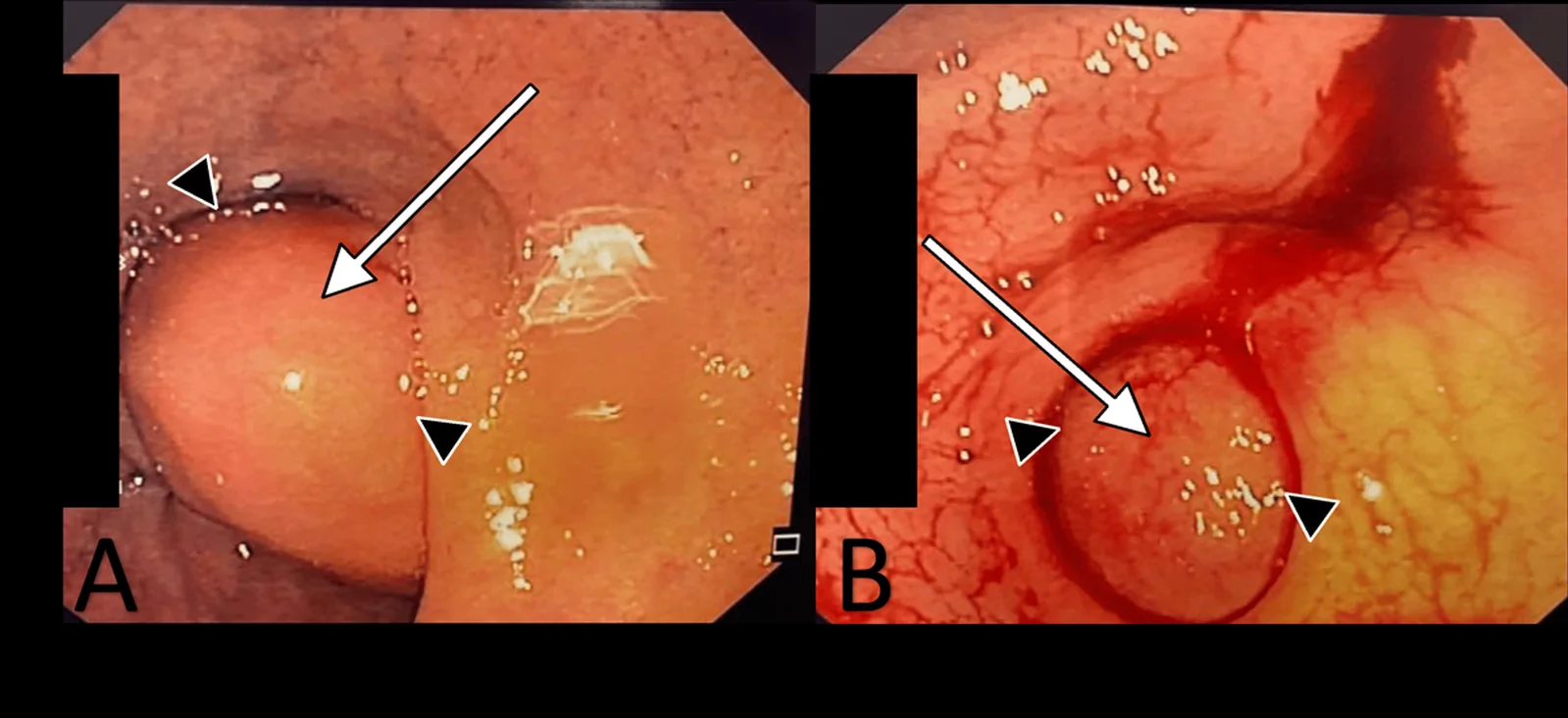
Upper endoscopic appearance of the duodenal lipoma (A) Retroflexed-free forward view of the distal first portion of the duodenum (D1) showing a broad-based, smooth-surfaced polypoid mass (white arrow) with intact, non-ulcerated overlying mucosa of normal color. Black arrowheads indicate the demarcated base of the lesion at its junction with the normal duodenal wall, confirming the broad (> 1 cm) attachment. The asterisk marks the compressed residual lumen, through which the endoscope was passed. (B) Closer view of the same lesion extending into the proximal second portion (D2). The mass (white arrow) is well circumscribed, with a soft, rounded contour and a clearly defined mucosal reflection at its margin (black arrowheads). Prominent overlying submucosal vessels and a small amount of contact bleeding from prior biopsy are visible superiorly. The lesion obstructed approximately 75% of the luminal circumference. When probed with closed biopsy forceps, the lesion indented easily and re-expanded slowly on release (positive "pillow sign")

A 40-year-old male smoker with hypertension presented with fatigue, postprandial epigastric discomfort, and recurrent melena. His hemoglobin was 9 g/dL (reference range: 13.5-17.5 g/dL in men), with a normal mean corpuscular volume (MCV) of 90 fL (reference range: 80-100 fL). Iron studies were normal. The serum iron level was 120 µg/dL (reference range: 60-170 µg/dL), and the ferritin level was 120 ng/mL (reference range: 30-400 ng/mL in men). The carcinoembryonic antigen (CEA) level was 2 ng/mL (reference range: < 5 ng/mL in smokers), and the carbohydrate antigen 19-9 (CA 19-9) level was 4 U/mL (reference range: < 37 U/mL). He had a three-month history of early satiety, limiting himself to pureed foods and soups, which was associated with a 10 kg weight loss and infrequent bowel movements (less than once a week). There was no family history of gastrointestinal disease or other obvious risk factors. Upper endoscopy identified a 4-5 cm, broad-based (> 1 cm) polypoid mass arising from the distal first portion of the duodenum (D1) and extending into proximal D2, obstructing approximately 75% of the lumen (Figures 1A, 1B).

**Figure 2:**
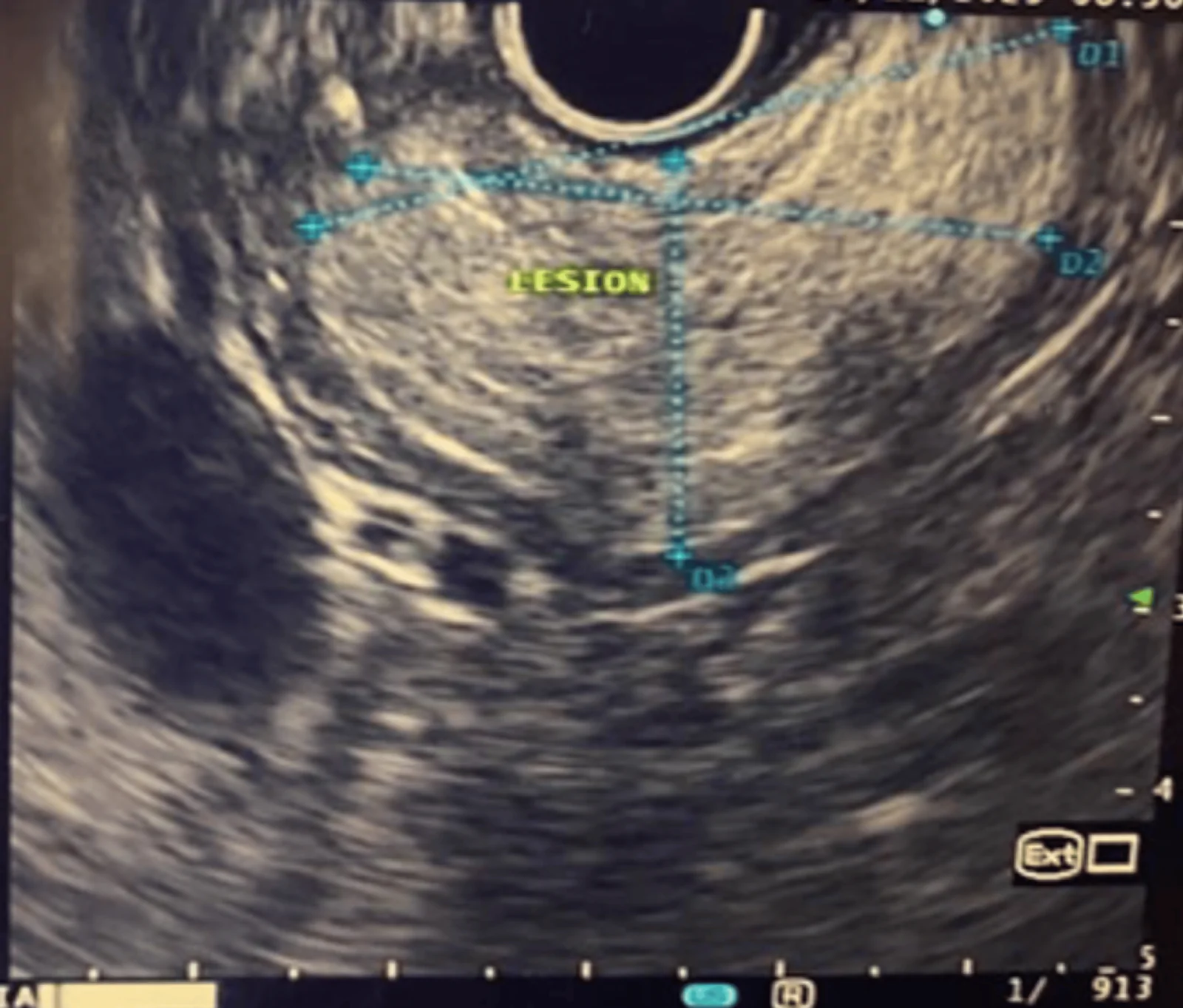
EUS showing a homogeneous, hyperechoic, well-defined submucosal mass measuring 5 × 3 × 2 cm, without regional adenopathy EUS: endoscopic ultrasound

The lesion was smooth, soft, and indented when probed with closed forceps, producing a positive "pillow sign," but did not show fat on biopsy. Forceps histology showed only hyperplastic mucosa with normal surface villi and no dysplasia. This non-diagnostic result reflects a recognized limitation of superficial biopsy, which samples the mucosa rather than the deeper submucosal fat [9]. EUS demonstrated a homogeneous, hyperechoic, well-defined submucosal mass measuring 5 × 3 × 2 cm, arising from the third echolayer (submucosa) and occupying 80% of the lumen, without regional adenopathy (Figure 2), a pattern characteristic of a lipoma [10].

**Figure 3:**
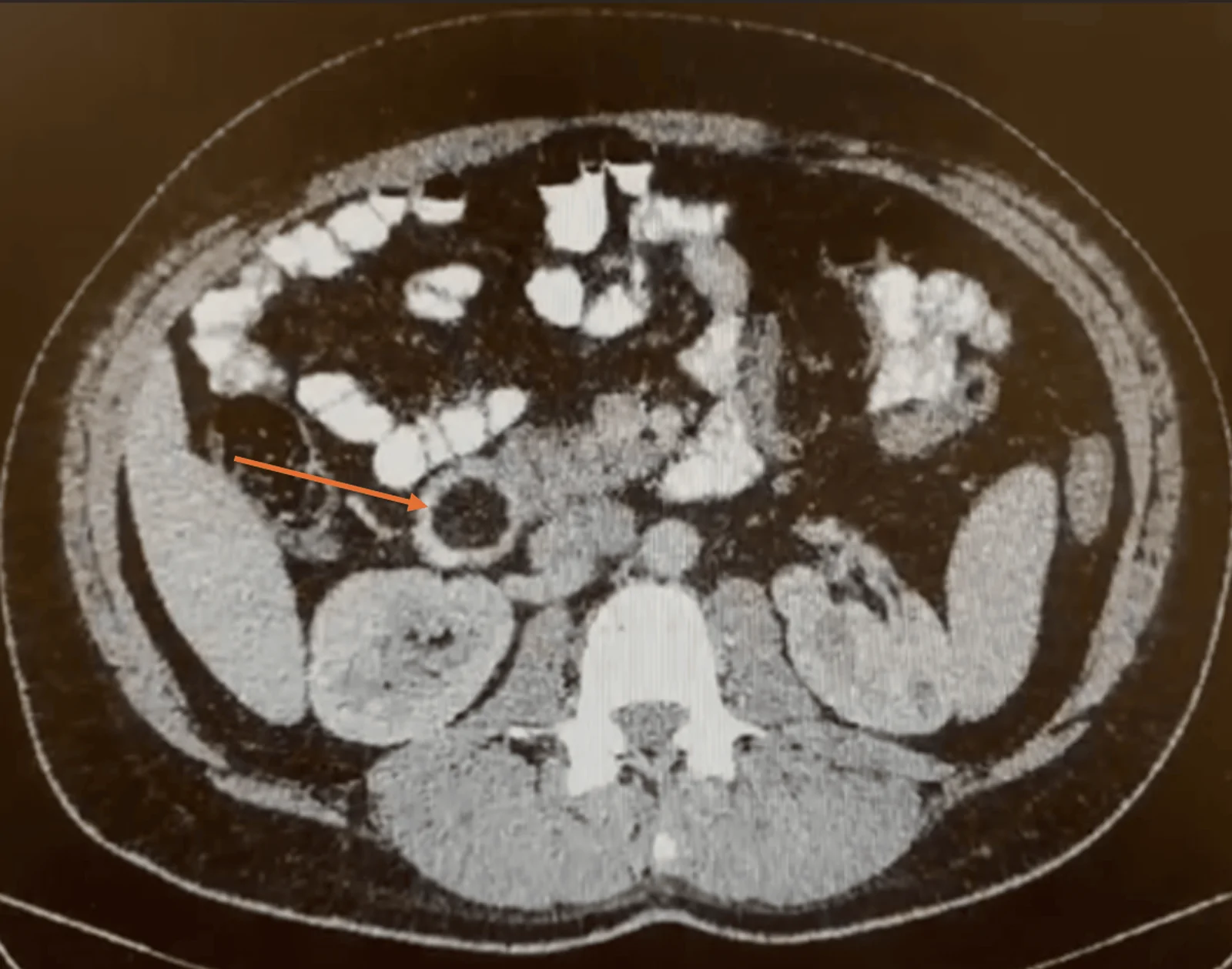
CT showing a solitary, well-defined, fat-density mass in the duodenum causing partial luminal obstruction CT: computed tomography

Contrast-enhanced CT confirmed a solitary, well-defined, homogeneous intraluminal mass with fat attenuation, with a mean attenuation of −95 HU (range: −80 to −110 HU). The lesion showed no post-contrast enhancement, thickened internal septa, soft-tissue nodularity, or calcification. It caused partial duodenal obstruction, with proximal gastric and D1 distension (Figure 3).

The patient was scheduled for open surgical excision because endoscopic resection was deemed risky due to the lesion's size and broad base ranging from 4 to 5 cm, which increased the risk of haemorrhage, perforation, and incomplete excision [7, 8].

Operative technique

Under general anesthesia, a 4-cm supraumbilical midline incision was made, and the peritoneal cavity was entered. A Kocher maneuver was performed to mobilize the duodenum and expose its posterior surface, confirming by palpation a mobile intraluminal mass arising from distal D1 and extending into D2, well proximal to the ampulla of Vater. A 2-cm longitudinal duodenotomy was made in the anterior wall opposite the lesion. The pedunculated submucosal mass was delivered through the duodenotomy and excised at its point of attachment. Because the lesion originated well proximal to the papilla, the ampulla, common bile duct, and pancreatic duct did not require instrumentation and were preserved, as were the surrounding duodenal mucosa and wall. The duodenotomy was closed transversely with 3-0 Vicryl sutures to minimize the risk of luminal narrowing, and the abdomen was closed in layers.

**Figure 4:**
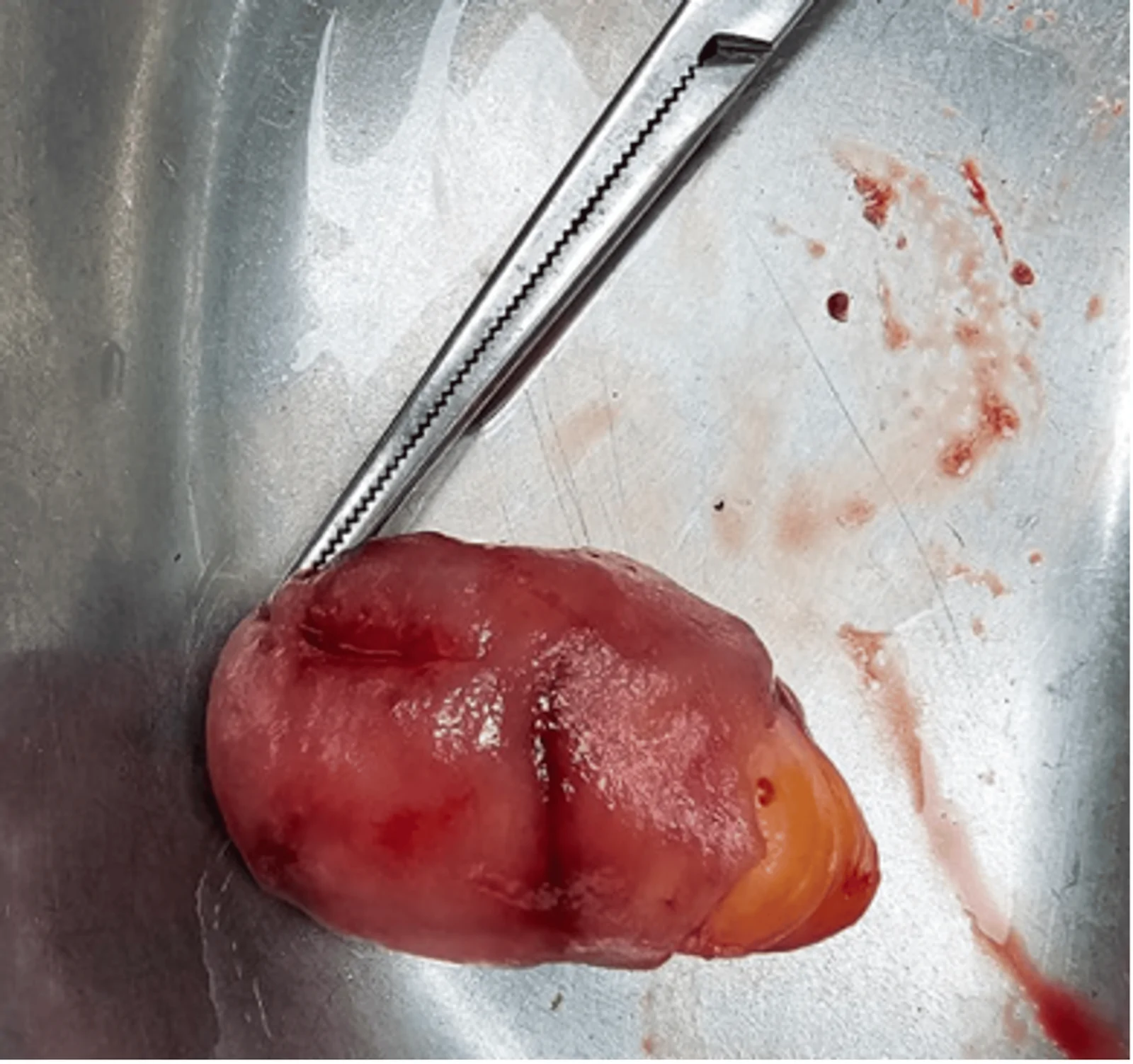
Gross appearance of the resected specimen The image shows a well-circumscribed, lobulated, yellow-tan submucosal mass

The gross specimen was a well-circumscribed, lobulated, yellow mass (Figure 4).

**Figure 5:**
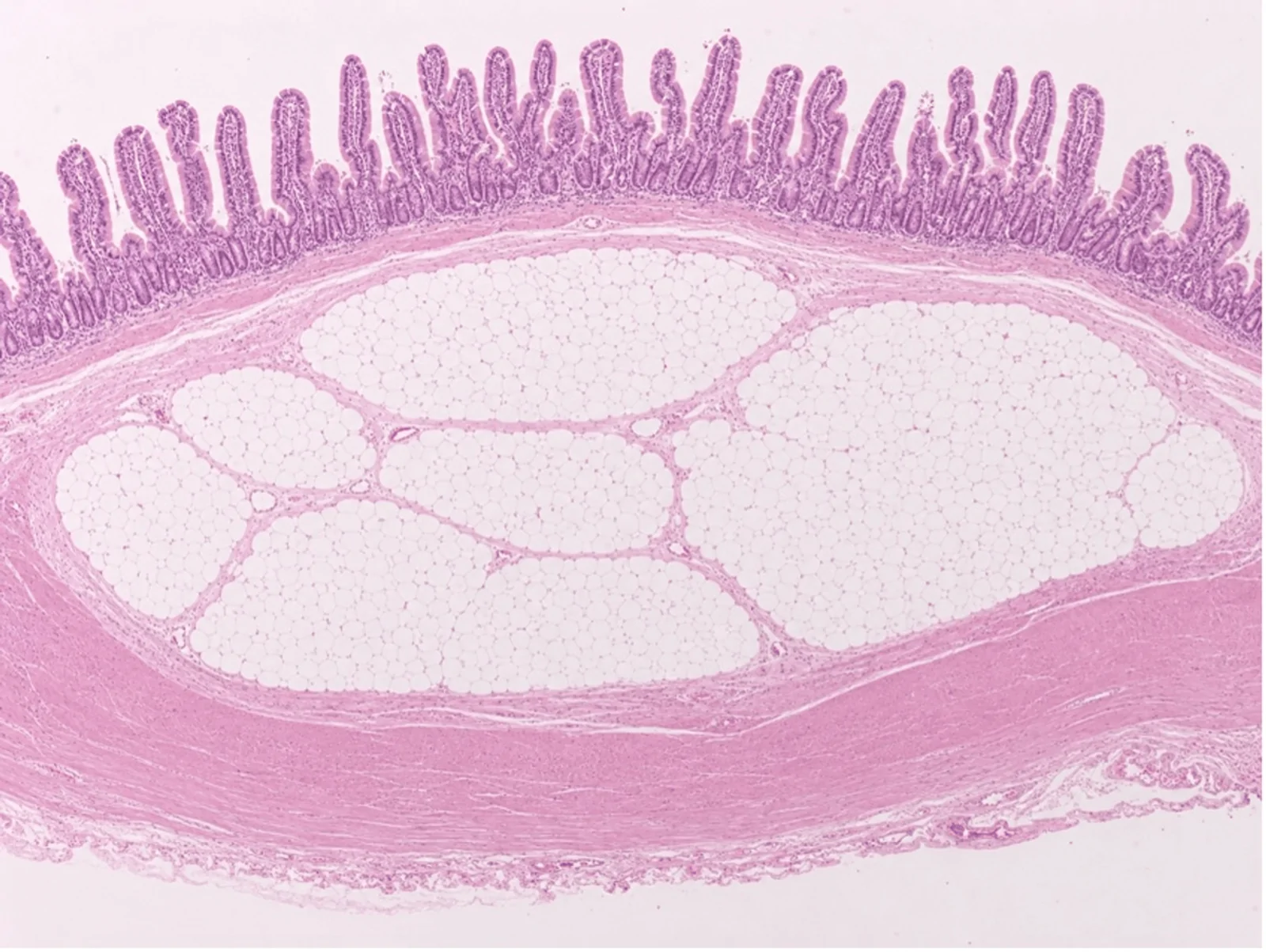
Photomicrograph (hematoxylin and eosin) showing mature adipose tissue within the submucosa beneath an intact, villous mucosa (consistent with submucosal lipoma)

Histopathology confirmed a submucosal lipoma composed of mature adipocytes beneath intact villous mucosa, without atypia (Figure 5).

The patient recovered uneventfully and was discharged on postoperative day three. At the five-month follow-up, his anemia had resolved, with hemoglobin rising from 9 g/dL preoperatively to 14.4 g/dL (reference range: 13.5-17.5 g/dL in men) and an MCV of 94 fL (reference range: 80-100 fL). Melena had not recurred postoperatively, and stool testing for occult blood was negative. Epigastric pain and early satiety had fully resolved, with the patient returning to an unrestricted solid diet, regaining 8 kg of the 10 kg lost, and normalizing his bowel habits to daily movements. There was no clinical evidence of recurrence or duodenal stricture at five months.

## Discussion

Duodenal lipomas are rare, accounting for only about 4% of gastrointestinal lipomas, the majority of which are located in the colon [9,11]. They are typically solitary, slow-growing, and most often located in D2 [1]. Lesions smaller than 2 cm are usually silent, while larger ones can cause postprandial pain, early satiety, anemia, or melena from mucosal ulceration, and rarely cause obstruction or intussusception [3]. Our patient's near-obstructive symptoms, marked weight loss, and prolonged dietary restriction represent an unusual advanced presentation for a benign tumor, illustrating how a slow-growing lipoma can mimic malignancy or a motility disorder until diagnosis.

There is no single completely diagnostic modality. In addition to defining the size, base, and position, endoscopy can indicate a lipoma based on the pillow/cushion sign and, less consistently, the "naked fat sign," when yellow fat is expressed through biopsy forceps [5]. This was not seen in this case because a routine pinch biopsy sampled only the mucosa, a known limitation [6,9]. Although it can be challenging to differentiate a lipoma from a Brunner gland hamartoma, EUS adds the characteristic finding of a submucosal, uniform, hyperechoic pattern that is highly predictive of lipoma [6,12]. By demonstrating a clearly defined mass with fat-equivalent attenuation, usually −70 to −120 HU, CT further supports the diagnosis [13]. Combining endoscopy, EUS, and CT reduces the need for repeated or deep biopsies by accurately distinguishing a lipoma from ectopic pancreas, gastrointestinal stromal tumors, and other subepithelial lesions [6,12,13].

Table 1 lays out the diagnostic modalities and key findings in the present case.

**Table 1:** Diagnostic modalities and key findings in the present case EGD: esophagogastroduodenoscopy; EUS: endoscopic ultrasound; CT: computed tomography; D1/D2: first/second portion of duodenum; GIST: gastrointestinal stromal tumor

Modality	Key findings	Diagnostic role and limitations
EGD	4-5 cm broad-based polypoid mass, distal D1 into proximal D2, obstructing ~75% of lumen; positive "pillow sign"; forceps biopsy non-diagnostic (hyperplastic mucosa only)	Defines size, base width, and location; pillow/cushion sign and naked fat sign suggest lipoma but are inconsistent; pinch biopsy samples mucosa only and often misses submucosal fat
EUS	Homogeneous, hyperechoic, well-defined submucosal mass, 5×3×2 cm, occupying ~80% of lumen; no regional adenopathy	Submucosal, homogeneous, hyperechoic pattern highly suggestive of lipoma; can be difficult to distinguish from Brunner gland hamartoma
CT	Solitary, well-defined, fat-density mass causing partial duodenal obstruction	Confirms fat-equivalent attenuation (typically -70 to -120 HU); complements endoscopy and EUS to help exclude GIST, ectopic pancreas, and other subepithelial lesions

Asymptomatic, incidentally discovered lipomas can be observed [3]. Endoscopic procedures such as snare polypectomy, endoloop-assisted unroofing, ESD, or EFTR are increasingly used to treat symptomatic lesions that are technically favorable, particularly when they are smaller, pedunculated, and have a narrow stalk [7,14,15,16]. These techniques avoid open surgery but carry a risk of bleeding, perforation, and incomplete or fragmented resection as lesion size and base width increase [8,17].

Our patient's lesion had characteristics that have been linked in published series to endoscopic failure: it was 4-5 cm in size, had a short, broad stalk with a base greater than 1 cm, occupied 75-80% of the duodenal lumen, and was located between distal D1 and proximal D2. A retrospective review of duodenal ESD found that a size greater than 40 mm, location at the duodenal flexure, and circumferential involvement greater than 50% were independent predictors of technical difficulty, all of which were present in our patient [8]. A recent review of published duodenal lipomas similarly found that, although no universal size cutoff has been defined, surgery was consistently favored over endoscopy for large, broad-based, or sessile lesions and for those close to the ampulla of Vater [18]. Due to the patient's lesion size, broad base, and nearly obstructive characteristics, with the lesion pedunculating from proximal D1 and extending distally, it was considered unsuitable for safe removal by endoscopic dissection or snare.

Instead of pancreas-sparing duodenectomy, segmental resection, or pancreaticoduodenectomy, local transduodenal excision through duodenotomy was adequate due to the benign nature of the tumor and its pedunculated attachment well proximal to the ampulla. This preserved the entire duodenal wall, ampulla, and biliary and pancreatic ducts, avoided an anastomosis, and allowed rapid recovery with a lower risk of bleeding, in line with findings that when a lesion can be delivered through the lumen without disturbing the papilla, a straightforward duodenotomy and excision are sufficient [3,18].

The choice between observation, endoscopic resection, and surgery for duodenal lipoma should be based on the patient's symptoms, lesion size and base width, morphology, layer of origin, and proximity to the ampulla and pancreaticobiliary structures. Open or minimally invasive transduodenal excision remains a safe, efficient, and suitably conservative choice when a lesion exceeds the technical limitations of safe endoscopic excision.

## Conclusions

Large or broad-based lesions can produce significant symptoms and are not necessarily appropriate for endoscopic treatment, while duodenal lipomas are uncommon and typically incidental findings. When endoscopic resection is risky or unlikely to be successful, transduodenal excision provides a safe, organ-preserving alternative, highlighting the importance of individualized, multidisciplinary decision-making. A multimodal work-up using endoscopy, EUS, and CT enables an informed choice of treatment.

## References

[1] Yang B, Jiang F, Lu P, Han H (2021). Minimally invasive management of large duodenal lipoma: endoscopic submucosal dissection. J Int Med Res.

[2] Wilson JM, Melvin DB, Gray G, Thorbjarnarson B (1975). Benign small bowel tumor. Ann Surg.

[3] Pei MW, Hu MR, Chen WB, Qin C (2017). Diagnosis and treatment of duodenal lipoma: a systematic review and a case report. J Clin Diagn Res.

[4] Dangoor A, Seddon B, Gerrand C, Grimer R, Whelan J, Judson I (2016). UK guidelines for the management of soft tissue sarcomas. Clin Sarcoma Res.

[5] Culver EL, McIntyre AS (2011). Sporadic duodenal polyps: classification, investigation, and management. Endoscopy.

[6] Gaspar JP, Stelow EB, Wang AY (2016). Approach to the endoscopic resection of duodenal lesions. World J Gastroenterol.

[7] Ren Z, Lin SL, Zhou PH, Cai SL, Qi ZP, Li J, Yao LQ (2019). Endoscopic full-thickness resection (EFTR) without laparoscopic assistance for nonampullary duodenal subepithelial lesions: our clinical experience of 32 cases. Surg Endosc.

[8] Kato M, Sasaki M, Mizutani M (2019). Predictors of technical difficulty with duodenal ESD. Endosc Int Open.

[9] Hwang JH, Saunders MD, Rulyak SJ, Shaw S, Nietsch H, Kimmey MB (2005). A prospective study comparing endoscopy and EUS in the evaluation of GI subepithelial masses. Gastrointest Endosc.

[10] Nakamura S, Iida M, Suekane H, Matsui T, Yao T, Fujishima M (1991). Endoscopic removal of gastric lipoma: diagnostic value of endoscopic ultrasonography. Am J Gastroenterol.

[11] Mayo CW, Pagtalunan RJ, Brown DJ (1963). Lipoma of the alimentary tract. Surgery.

[12] Figueiredo PC, Pinto-Marques P, Mendonça E, Oliveira P, Brito M, Serra D (2013). Duodenal subepithelial hyperechoic lesions of the third layer: not always a lipoma. World J Gastrointest Endosc.

[13] Waligore MP, Stephens DH, Soule EH, McLeod RA (1981). Lipomatous tumors of the abdominal cavity: CT appearance and pathologic correlation. AJR Am J Roentgenol.

[14] Blanchet MC, Arnal E, Paparel P, Grima F, Voiglio EJ, Caillot JL (2003). Obstructive duodenal lipoma successfully treated by endoscopic polypectomy. Gastrointest Endosc.

[15] Huang WH, Peng CY, Yu CJ, Chou JW, Feng CL (2008). Endoloop-assisted unroofing for the treatment of symptomatic duodenal lipomas. Gastrointest Endosc.

[16] Wu C, Yang JF, Tan Q, Zhang Q, Hu B (2017). En bloc resection of a large symptomatic duodenal lipoma by endoscopic submucosal dissection. VideoGIE.

[17] Yu HG, Ding YM, Tan S, Luo HS, Yu JP (2007). A safe and efficient strategy for endoscopic resection of large, gastrointestinal lipoma. Surg Endosc.

[18] Potums L, Huysentruyt F, D'hooge P, Delvaux P (2025). Giant duodenal lipoma: a case report and narrative review of published cases and their treatment strategy. Eur J Gastroenterol Digestive Dis.

